# Trends and Disparities in Maternal Self-Reported Mental and Physical Health

**DOI:** 10.1001/jamainternmed.2025.1260

**Published:** 2025-05-27

**Authors:** Jamie R. Daw, Colleen L. MacCallum-Bridges, Lindsay K. Admon

**Affiliations:** 1 Department of Health Policy and Management, Columbia University Mailman School of Public Health, New York, New York; 2 Department of Obstetrics and Gynecology, University of Michigan, Ann Arbor; 3 Institute for Healthcare Policy and Innovation, University of Michigan, Ann Arbor

## Abstract

**Importance:**

While rising maternal morbidity and mortality have been well documented in the US, national data on general maternal physical and mental health beyond the perinatal period are lacking.

**Objective:**

To examine recent trends and disparities in self-reported maternal physical and mental health.

**Design, Setting, and Participants:**

In this pooled, cross-sectional study, the nationally representative National Survey of Children’s Health was used to assess self-reported mental and physical health of female biological or adoptive parents of children aged 0 to 17 years (hereafter, mothers) from 2016 to 2023.

**Main Outcomes and Measures:**

The main outcomes were self-reported maternal physical and mental health, measured on a 4-point Likert scale (excellent, very good, good, fair/poor). Regression models were used to estimate trends and sociodemographic disparities in self-reported physical and mental health.

**Results:**

A total of 198 417 mothers (mean [SD] age, 39.0 [0.04] years) were included in the sample, representing 42 130 370 individuals nationally in the weighted sample. Among mothers in the study, 89.8% were 30 years and older; 57.9% of mothers had a privately insured child, 35.7% had a publicly insured child, and 6.4% had an uninsured child. The unadjusted prevalence of excellent physical health declined from 28.0% to 23.9%, while good physical health rose from 24.3% to 28.1%, and fair/poor physical health did not significantly change. The unadjusted prevalence of excellent mental health declined from 38.4% to 25.8%, while good mental health rose from 18.8% to 26.1%, and fair/poor mental health rose from 5.5% to 8.5%. After adjusting for secular changes in the sociodemographic characteristics of mothers, excellent physical health decreased by 4.2 percentage points (pp) (95% CI, −5.7 to −2.8 pp), excellent mental health decreased by 12.4 pp (95% CI, −14.0 to −10.7 pp), and fair/poor mental health increased by 3.5 pp (95% CI, 2.6-4.4 pp) over the 8-year study period. Mental health declines occurred broadly across socioeconomic subgroups. However, self-reported physical and mental health status was significantly lower among mothers who were US born, single parents, less educated, and those with publicly insured or uninsured children.

**Conclusions and Relevance:**

In this cross-sectional study, self-reported mental health was found to have significantly declined for female parents. Investments are needed to investigate and address the underlying causes of mental health decline among US mothers, especially those of low socioeconomic status.

## Key Points

**Question:**

What are the recent national trends in self-reported maternal physical and mental health?

**Findings:**

In this cross-sectional study of 198 417 female parents of children aged 0 to 17 years, large declines in self-reported mental health and small declines in self-reported physical health from 2016 to 2023 were observed. Mental health declines occurred across all socioeconomic subgroups; however, mental and physical health status was significantly lower for single female parents, those with lower educational attainment, and those with publicly insured children.

**Meaning:**

Investments are needed to investigate and address the underlying causes of mental health decline among US mothers, particularly for those of low socioeconomic status.

## Teaser

This cross-sectional study examines trends and disparities in self-reported maternal physical and mental health between 2016 and 2023 in the US.

## Introduction

The maternal health crisis in the US has largely been defined by recent increases in maternal mortality and stark inequities in pregnancy-related deaths by race, geography, and socioeconomic status.^1,2,3,4^ In August 2024, the US Surgeon General issued an advisory, “Parents Under Pressure,” identifying parental mental health and well-being as another critical public health challenge requiring immediate national attention.^5^ The advisory highlights rising levels of parental stress and the intergenerational public health impact of poor health among US parents. The report offers a new perspective on recent increases in maternal morbidity and mortality as part of a broader crisis affecting parental health beyond the perinatal period.

Despite its critical importance, few studies have examined the general health status of mothers in the US, and those that have focused on specific subpopulations, such as mothers of young children^6^ or children with special health care needs.^7^ While the recent Surgeon General’s report compiles data on rising levels of stress and certain physical and mental health conditions among mothers, it also underscores the need for more comprehensive research on the health and well-being of US parents more broadly. Using nationally representative data on US children, this study aims to address this gap by examining recent national trends and disparities in self-reported physical and mental health among female parents.

## Methods

The reporting of this cross-sectional study followed the Strengthening the Reporting of Observational Studies in Epidemiology (STROBE) reporting guideline. This study of public-use data was deemed exempt by the University of Michigan institutional review board. We used data from 2016 to 2023 from the National Survey of Children’s Health (NSCH), an annual nationally representative survey of households with children aged 0 to 17 years.^8^ The survey is completed by a parent or other adult caregiver. From 2016 to 2023, 61% of the NSCH respondent parents/caregivers were biological, step, foster, or adoptive female parents; 27% were biological, step, foster, or adoptive male parents; 8% were other relatives or caregivers; and 4% were of unknown relation or sex. The survey captures data on child demographics, health, and health care, as well as the demographics and health of up to 2 caregivers, as reported by the respondent parent/caregiver. Additional information on NSCH methods is available from the US Census Bureau.^8^

We selected a study period of 2016 to 2023 because 2016 was the first year of the modernized NSCH, and 2023 is the most recent year of data available. Because we were interested in measuring self-reported maternal health, we limited the sample to respondents who were biological or adoptive female parents (hereafter, mothers).

### Measures

We measured 2 primary outcomes, self-reported physical and mental maternal health, based on the survey items: (1) “In general, how is your physical health?” and (2) “In general, how is your mental or emotional health?” measured on a 5-point Likert scale (excellent, very good, good, fair, or poor). We operationalized these outcomes either as a 4-point scale, combining fair or poor due to the low prevalence of poor health ratings (<1.1% for both physical and mental health), or as a binary measure indicating self-reported fair or poor health (vs excellent, very good, or good).

We identified sociodemographic characteristics self-reported in the NSCH that may be correlated with maternal health, including maternal age, education, and nativity; family structure; and the child’s age, race, ethnicity, and health insurance status. We created a combined race and ethnicity variable from separate Hispanic ethnicity and race questions.^9^ We combined Asian with Native Hawaiian and Pacific Islander due to small sample sizes; other race and ethnicity categories are as defined on the NSCH questionnaire.

### Statistical Analysis

To assess temporal trends, we plotted the weighted prevalence of self-reported physical and mental health ratings by year. Then, we used unadjusted and adjusted generalized ordinal logistic regression models to estimate the total percentage point (pp) change and the annual pp change (ie, the annual trend) in the prevalence of the 4-point scale ratings between 2016 and 2023. In addition to estimating absolute pp changes, we also converted pp changes to relative percentage changes from the baseline year of 2016. Adjusted models included sociodemographic characteristics to account for compositional changes over the study period.

We conducted 4 secondary analyses. First, to assess whether the onset of the COVID-19 pandemic was associated with changes in maternal health levels or trends, we conducted a segmented regression analysis of 2016 to 2019 compared with 2020 to 2022 (omitting 2023 as this year marked the end of the COVID-19 public health emergency).^10^ Second, to identify whether changes in fair or poor self-reported health varied across specific populations, we measured trends stratified by sociodemographic subgroups. Third, to identify disparities in maternal self-reported fair or poor health, we used the pooled data (2016-2023) and unadjusted and adjusted logistic regression models to estimate odds ratios between sociodemographic subgroups. We selected reference groups to be either the largest group (eg, for child age, school-aged children) or the group with the greatest social or economic privilege (eg, for education, bachelor’s degree or more). Finally, to provide context and comparison for the results among female parents, we examined trends in self-reported physical and mental health for male parents following the same inclusion criteria and methods for female parents.

To ensure a consistent sample across all analyses, we conducted a complete case analysis, excluding respondents with missing outcome or covariate data. Missingness was relatively rare (<5%); thus, we did not impute missing values.^11^ We conducted analyses in Stata/SE 18 (StataCorp LLC) using NSCH sampling weights to generate nationally representative estimates that account for the survey design and nonresponse. We considered 2-sided *P* values less than .05 to be statistically significant.

## Results

### Sample Characteristics

From 2016 to 2023, the NSCH included 334 708 respondent households with children aged 0 to 17 years. Of these, 203 818 mothers were identified, of whom 198 417 (97.4%) had complete information on the study outcomes and covariates (mean [SD] age, 39.0 [0.04] years), representing 42 130 370 individuals nationally in the weighted sample. Among mothers in the study, 89.8% were 30 years and older; 57.9% of mothers had a privately insured child, 35.7% had a publicly insured child, and 6.4% had an uninsured child. The racial and ethnic distribution of the children was as follows: 0.6% were American Indian or Alaska Native; 27.2% were Hispanic; 3.4% were non-Hispanic Asian, Native Hawaiian, or Pacific Islander; 12.3% were non-Hispanic Black; 51.1% were non-Hispanic White; and 5.4% were non-Hispanic multiracial. In addition, 77.2% of mothers lived in a 2-parent household, 78.8% were US born, and 72.8% had some education beyond high school (Table 1).

**Table 1.** Sample Characteristics

Characteristic	No. (weighted %)		
	2016-2023 (N = 198 417)	2016 (n = 30 068)	2023 (n = 32 416)
Child age, y			
Infants, <1	7079 (4.9)	1184 (5.5)	1160 (4.7)
Toddlers/preschool age, 1-5	60 280 (27.2)	7790 (27.5)	11 689 (26.5)
School age, 6-12	68 141 (39.5)	10 673 (39.7)	10 494 (39.4)
Adolescents, 13-17	62 917 (28.4)	10 421 (27.3)	9073 (29.4)
Parent-reported child race and ethnicity			
Hispanic	26 771 (27.2)	3255 (26.3)	4897 (28.8)
Non-Hispanic American Indian or Alaska Native	1003 (0.6)	155 (0.6)	158 (0.6)
Non-Hispanic Asian or Native Hawaiian and Pacific Islander	7980 (3.4)	1205 (3.4)	1438 (3.6)
Non-Hispanic Black	11 658 (12.3)	1689 (13.0)	1794 (11.4)
Non-Hispanic White	137 441 (51.1)	21 973 (52.1)	21 741 (48.4)
Non-Hispanic multiracial	13 564 (5.4)	1791 (4.6)	2388 (7.2)
Child insurance status			
Private	140 332 (57.9)	22 206 (56.0)	22 726 (58.7)
Any public	49 851 (35.7)	6617 (37.4)	8399 (35.2)
Uninsured	8234 (6.4)	1245 (6.6)	1291 (6.1)
Maternal age, y			
18-24	3152 (2.1)	729 (3.5)	446 (1.8)
25-29	13 857 (8.0)	2211 (9.2)	2164 (6.3)
30-34	34 137 (18.5)	5071 (19.6)	5948 (18)
35-39	48 036 (25.8)	6500 (24.8)	8294 (25.0)
≥40	99 235 (45.5)	15 557 (42.9)	15 564 (49.0)
Maternal education			
<High school	6357 (11.9)	850 (12.8)	1025 (11.2)
High school diploma	21 422 (15.3)	3197 (15.6)	3284 (15.1)
Some college^a^	58 270 (28.2)	9346 (30.2)	8649 (25.5)
≥Bachelor’s degree	112 368 (44.6)	16 675 (41.4)	19 458 (48.1)
Maternal nativity			
US born	173 665 (78.8)	26 704 (79.0)	28 064 (77.8)
Non-US born	24 752 (21.2)	3364 (21.0)	4352 (22.2)
Family structure			
2 Parents (male and female)	158 373 (75.8)	24 712 (77.9)	26 285 (76.3)
Single female parent	37 785 (22.8)	5122 (20.9)	5822 (22.7)
2 Parents (both female)	2259 (1.4)	234 (1.2)	309 (1.0)

^a^ Some college includes an associate’s degree or vocational training.

The demographic composition of the maternal population changed over the study period, including statistically significant increases in the proportion with non-Hispanic multiracial children (4.6% to 7.2% in 2023), privately insured children (56.0% to 58.7%), and those living in single parent households (20.9% to 22.7%). There were particularly large increases in mothers 40 years or older (42.9% in 2016 to 49.0% in 2023) and those with a bachelor’s degree or higher education (41.4% to 48.1%).

### Trends in Maternal Physical and Mental Health

**Figure 1.**
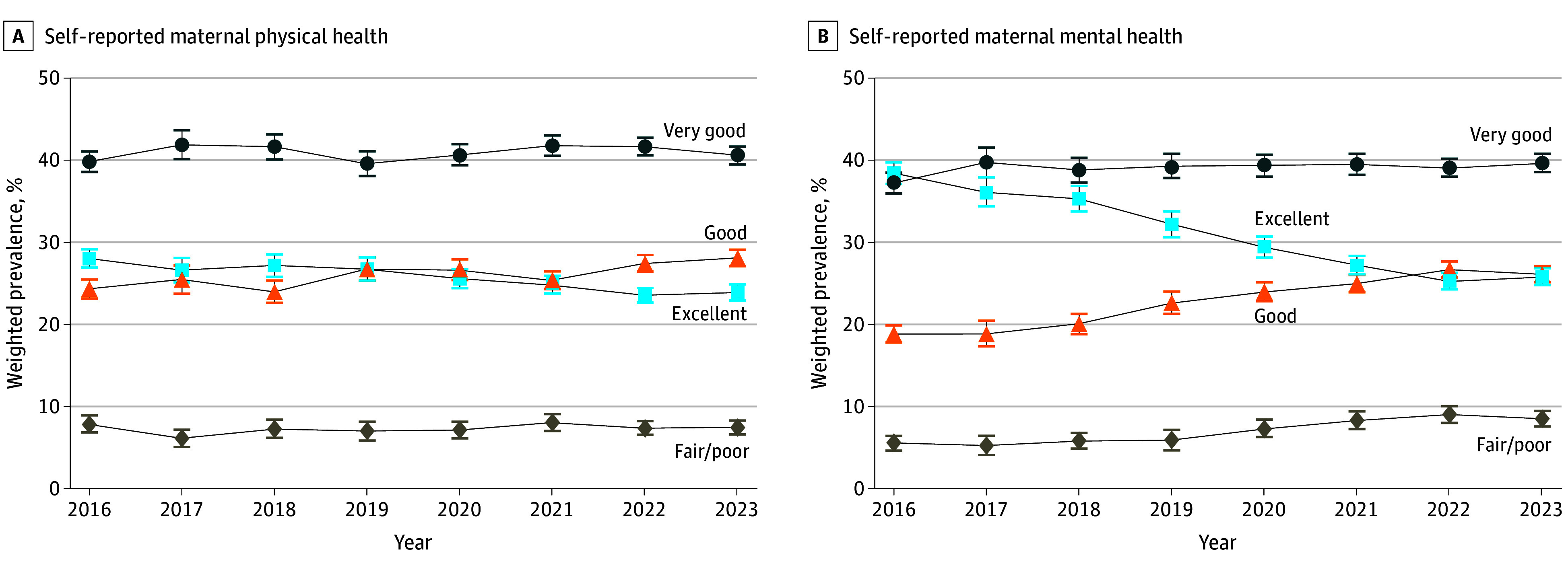
Trends in Self-Reported Maternal Physical and Mental Health, 2016-2023 The squares indicate the adjusted odds ratios, and the error bars indicate 95% CIs.

As shown in Figure 1, the unadjusted prevalence of excellent physical health declined from 28.0% in 2016 to 23.9% in 2023, corresponding with an increase in good physical health (24.3% to 28.1%). After adjusting for sociodemographic characteristics, excellent physical health decreased by 4.2 pp (95% CI, −5.7 to −2.8 pp) between 2016 and 2023 (annual trend: −0.6 pp per year [95% CI, −0.8 to −0.5 pp]) and good physical health increased by 4.4 pp (95% CI, 2.9-6.0). Fair/poor physical health did not significantly change (Table 2). Declines in mental health were more substantial. Excellent mental health declined from 38.4% to 25.8%, corresponding with increases in both good (18.8% to 26.1%) and poor/fair mental health (5.5% to 8.5%). After adjustment, excellent mental health declined by 12.4 pp (95% CI, −14.0 to −10.7 pp) from 2016 to 2023 (annual trend: −2.0 pp per year [95% CI, −2.2 to −1.8 pp]; Table 2), while good mental health increased by 7.5 pp (95% CI, 6.1-8.9 pp), and poor/fair mental health increased by 3.5 pp (95% CI, 2.6-4.4 pp).

**Table 2.** Annual Trends and Overall Prevalence Changes in Self-Reported Maternal Physical and Mental Health, 2016-2023

Outcome	Prevalence, % (95% CI)			Annual trend from 2016 to 2023, pp (95% CI)^a^		Prevalence change from 2016 to 2023, pp (95% CI)^a^		Adjusted change from 2016, %
	2016		2023	Unadjusted	Adjusted^b^	Unadjusted	Adjusted^b^	
**Physical health**								
Poor or fair	7.9 (7.1 to 8.7)	7.4 (6.8 to 8.1)		0.1 (−0.1 to 0.2)	0.1 (0 to 0.2)	−0.4 (−1.5 to 0.6)	−0.1 (−1.2 to 0.9)	1.3
Good	24.3 (23.1 to 25.5)	28.1 (27.1 to 29.1)		0.5 (0.3 to 0.7)	0.5 (0.4 to 0.7)	3.8 (2.2 to 5.4)	4.4 (2.9 to 6.0)	18.1
Very good	39.8 (38.6 to 41.1)	40.6 (39.5 to 41.7)		0.1 (−0.1 to 0.3)	0 (−0.2 to 0.2)	0.8 (−0.9 to 2.4)	−0.1 (−1.8 to 1.7)	−0.3
Excellent	28.0 (26.9 to 29.2)	23.9 (22.9 to 24.9)		−0.6 (−0.8 to −0.4)	−0.6 (−0.8 to −0.5)	−4.1 (−5.6 to −2.6)	−4.2 (−5.7 to −2.8)	−15.0
**Mental health**								
Poor or fair	5.5 (4.9 to 6.2)	8.5 (7.8 to 9.2)		0.6 (0.5 to 0.7)	0.7 (0.5 to 0.8)	3.0 (2.0 to 3.9)	3.5 (2.6 to 4.4)	63.6
Good	18.8 (17.8 to 19.9)	26.1 (25.1 to 27.1)		1.3 (1.1 to 1.4)	1.3 (1.1 to 1.5)	7.3 (5.9 to 8.7)	7.5 (6.1 to 8.9)	39.9
Very good	37.2 (36.0 to 38.5)	39.6 (38.5 to 40.7)		0.2 (0 to 0.4)	0.1 (−0.1 to 0.3)	2.4 (0.7 to 4.1)	1.4 (−0.4 to 3.1)	3.8
Excellent	38.4 (37.1 to 39.7)	25.8 (24.8 to 26.8)		−2.0 (−2.2 to −1.8)	−2.0 (−2.2 to −1.8)	−12.6 (−14.3 to −11.0)	−12.4 (−14.0 to −10.7)	−32.3

Abbreviation: pp, percentage point.

^a^ Prevalence changes and annual trends estimated using a generalized ordinal logistic model.

^b^ Adjusted model includes child age, child race and ethnicity, child insurance status, maternal age, maternal education, maternal nativity, and family structure.

Visual trends and the segmented regression results suggest that secular shifts from excellent to good physical and mental health originated prior to the COVID-19 pandemic. No significant changes in the prevalence level or trend in excellent, very good, or good physical or mental health associated with the onset of the pandemic were identified (eTable 1 in Supplement 1). However, there was a pandemic-related increase in the prevalence level (1.5 pp [95% CI, 0.3-2.6 pp]) and trend (0.6 pp [95% CI, 0-1.2 pp]) of fair/poor mental health.

Increases in fair/poor mental health occurred across nearly all sociodemographic subgroups, although the relative magnitude of the increase varied (eFigure 1, eTables 2-3 in Supplement 1). In adjusted models, rates of fair/poor mental health more than doubled from 2016 to 2023 for some subgroups including mothers of preschool-aged children (5.5 pp [95% CI, 4.0-7.0 pp]), non-Hispanic Asian or Native Hawaiian and Pacific Islander children (3.6 pp [95% CI, −0.3 to 7.4 pp]), and privately insured children (3.4 pp [95% CI, 2.6-4.3 pp]). In contrast, statistically significant increases in fair/poor physical health were limited to mothers of privately insured children (0.8 pp [95% CI, 0-1.5 pp]).

### Disparities in Maternal Physical and Mental Health

**Figure 2.**
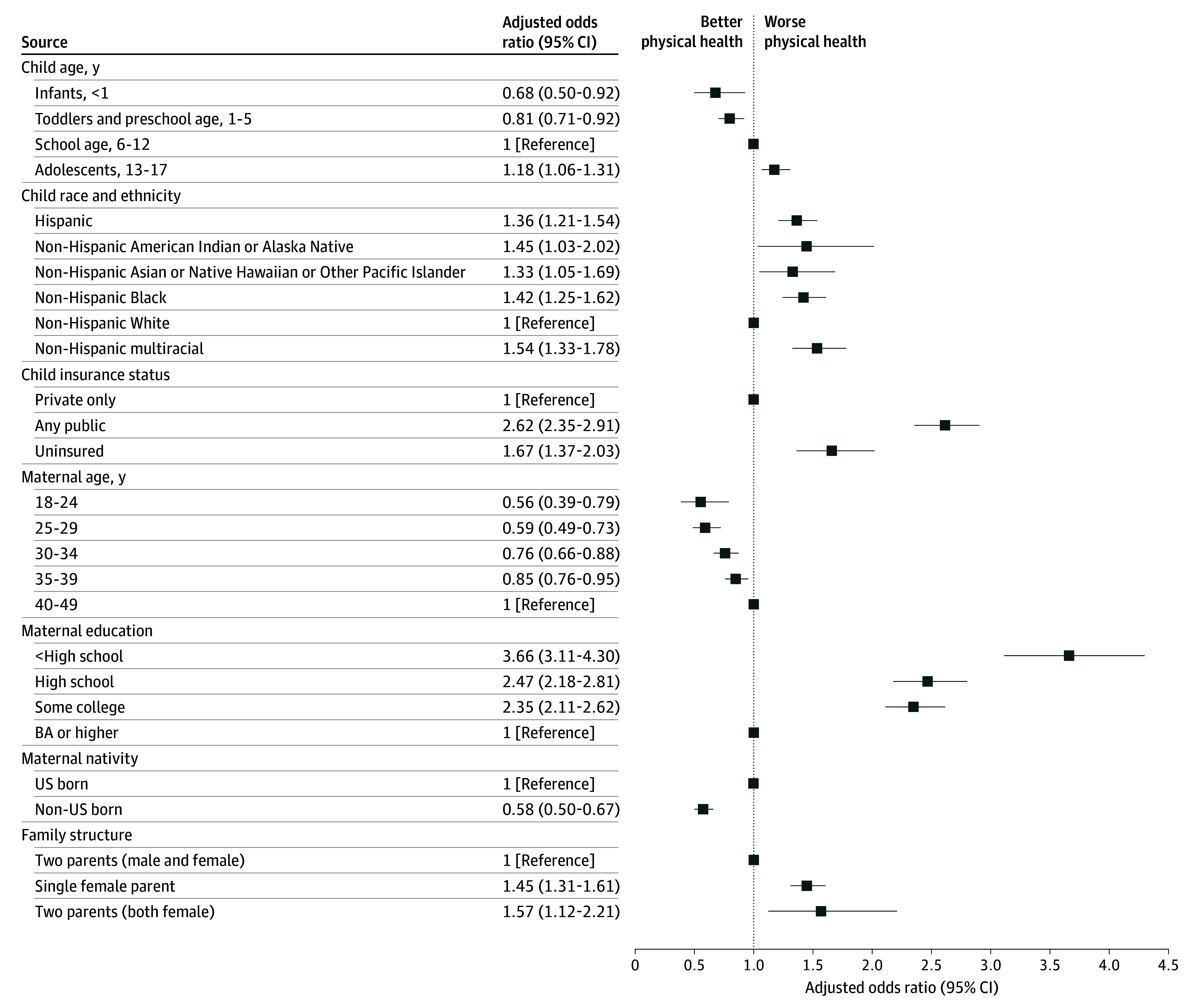
Adjusted Odds of Fair or Poor Maternal Physical Health by Sociodemographic Characteristics, 2016-2023 All estimates are presented in eTable 4 in Supplement 1. BA indicates bachelor of arts.

**Figure 3.**
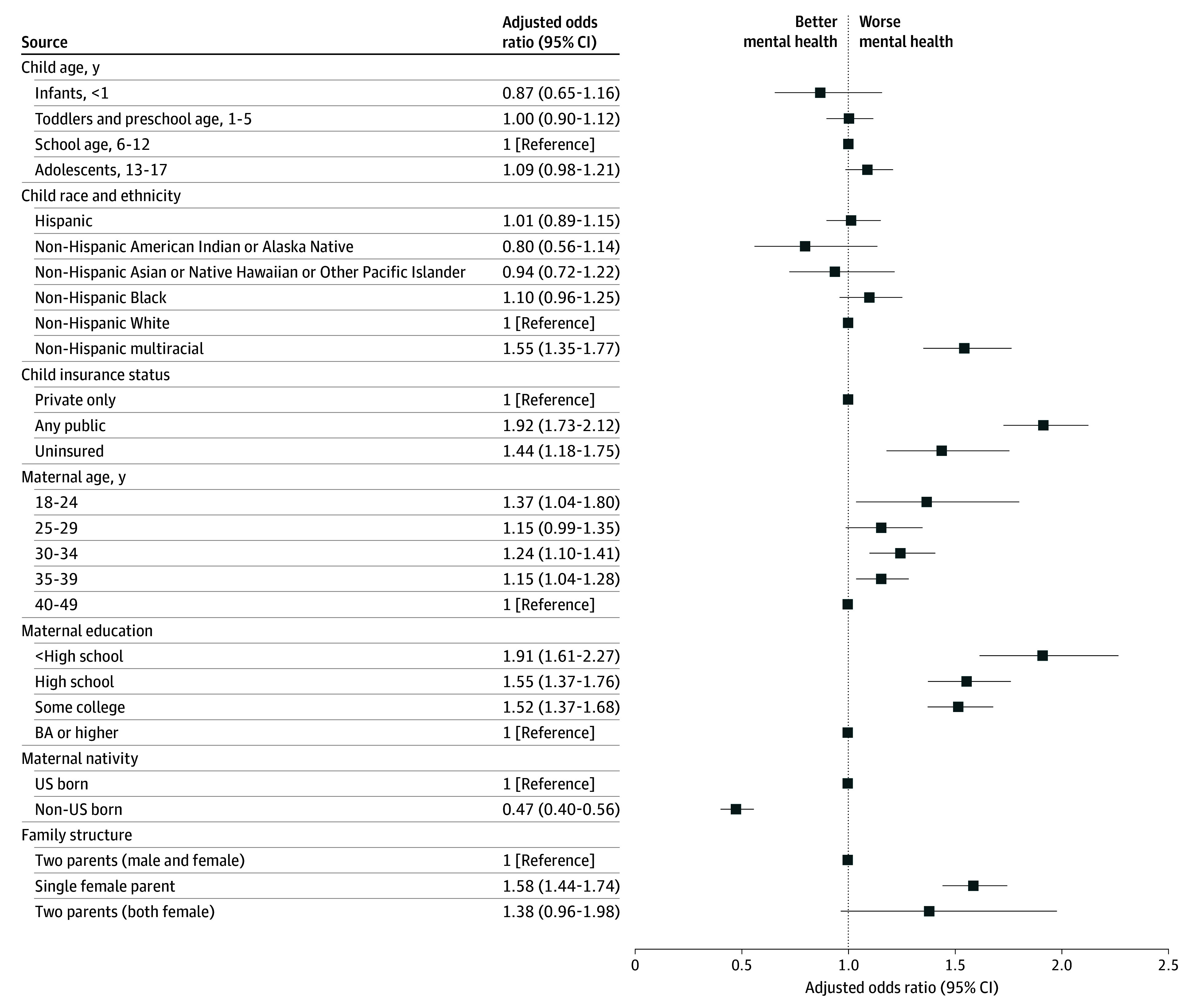
Adjusted Odds of Fair or Poor Maternal Mental Health by Sociodemographic Characteristics, 2016-2023 All estimates are presented in eTable 4 in Supplement 1. BA indicates bachelor of arts.

Using pooled data from 2016 to 2023, significantly worse physical and mental health status was observed among mothers of lower educational attainment, mothers with uninsured or publicly insured children, US-born mothers, and single parents (Figures 2 and 3; eTable 4 in Supplement 1). Disparities by education, insurance, nativity, and family structure were larger for physical than for mental health; for example, compared to mothers with a bachelor’s degree or higher, the adjusted odds of fair/poor physical health for mothers with less than high school education were 3.7 times (95% CI, 3.1-4.3) higher while the adjusted odds of fair/poor mental health were 1.9 times (95% CI, 1.6-2.3) higher.

Disparities by maternal age and child race and ethnicity were more nuanced. For physical health, a steep age gradient was observed for physical health (with higher odds of fair/poor physical health status for older groups) and wide racial disparities: all minoritized racial groups reported significantly higher rates of fair/poor physical health than non-Hispanic White individuals. For mental health, a reverse age gradient was observed (with worse adjusted mental health status for younger groups), and racial disparities were minimal.

### Trends in Paternal Physical and Mental Health

Among male parents, the unadjusted prevalence of excellent physical health declined from 29.9% in 2016 to 26.4% in 2023 (adjusted decrease: −3.9 pp [95% CI, −6.1 to −1.7 pp]) reflecting an increase in good (3.0 pp [95% CI, 0.9-5.1 pp]) and fair/poor physical health (1.8 pp [95% CI, 0.7-2.9 pp]; eFigure 2, eTable 5 in Supplement 1). Relative declines in mental health were similar in magnitude to female parents with a decrease in excellent mental health (−10.5 pp [95% CI, −12.8 to −8.1 pp]) and increase in good (6.7 pp [95% CI, 5.0-8.5 pp]) and fair/poor mental health (2.0 [95% CI, 1.1-2.8 pp]). Like female parents, while changes in self-reported fair/poor physical health were not associated with the onset of the COVID-19 pandemic, the segmented regression analysis revealed a significant change in the prevalence of fair/poor mental health (1.1 pp [95% CI, 0.2-2.0 pp]; eTable 6 in Supplement 1). Despite similar relative declines over the study period, male parents had better physical health status and substantially better mental health status during all years studied. In 2023, the prevalence of fair/poor mental health was 4.0 pp higher among female parents compared to male parents (8.5% compared to 4.5%), while fair/poor physical health was 1.5 pp higher in 2016 (7.4% compared to 5.9%).

## Discussion

In this cross-sectional analysis of national survey data, we found a substantial decline in the self-reported mental health of mothers of US children from 2016 to 2023. Over this period, the prevalence of self-reported poor or fair mental health increased by 3.5 pp, representing a 63.6% relative increase from the baseline prevalence of 5.5% in 2016. Increases in fair or poor mental health occurred across the socioeconomic spectrum. In contrast, physical health declines were smaller in relative magnitude and associated with shifts from excellent to good physical health, without a detectable change in poor or fair physical health. Downward trends in excellent maternal mental and physical health originated before the COVID-19 pandemic, and less than half of the increase in fair/poor mental health was estimated to coincide with the onset of the public health emergency.

Our findings are supportive of the claim made by some scholars that maternal mortality may be a canary in the coal mine for women’s health more broadly.^12,13^ Our results offer more specificity to this argument, highlighting the rising tide of worsening mental health among parenting women as a key target for efforts to improve maternal-child health in the US. Our results are consistent with documented increases in depression and anxiety diagnoses and suicidality among pregnant and reproductive-aged women, as well as the general US adult population.^14,15,16,17^ It is also consistent with rising mental health–related mortality including suicide, overdose, and their co-occurrence among reproductive-aged women^18^ and the documented principal causes of pregnancy-related mortality: maternal mental health conditions are now the leading cause of pregnancy-related death in the US, accounting for one-quarter (22.7%) of all deaths according to Maternal Mortality Review Committee data from 2017 to 2019.^1,19,20^

Our findings suggest that mothers may warrant special consideration in efforts to improve parental health, and mental health in particular. We found consistently worse health status among mothers compared to fathers, particularly for fair/poor mental health, which was reported by 1 in 12 female parents (8.5%) in 2023 compared to only 1 in 22 male parents (4.5%). However, we also identified concerning health declines among male parents that are closing this gender gap. Male parents had similar relative declines in mental health and increases in fair/poor physical health that were not observed among female parents. In 2023, the female-male parent disparity in fair/poor physical health narrowed to 1.5 pp (7.4% among female compared to 5.9% among male parents), a decline from 3.7 pp in 2016 (7.9% among female compared to 4.2% among male parents; a 88% relative disparity).

While prior research has found that mental health declines are occurring across the working-age population,^15,16,21^ as noted in the Surgeon General’s advisory, worsening mental health among parents has particularly important and broad implications for public health and society that deserve distinct attention.^5^ Mental health disorders are associated with other medical comorbidities, premature mortality, and worse social and economic outcomes.^22,23,24,25,26^ The mental health of parents can also have profound intergenerational impacts; for example, maternal mood disorders are associated with adverse birth outcomes, early child development, and long-term child mental and physical health.^5,27,28^ Parental mental health disorders can also contribute to social risk factors for children, such as exposure to substance use disorder, intimate partner violence, and other adverse childhood experiences.^27,29^ Our findings suggest that these potential consequences of poor maternal mental health fall most heavily on disadvantaged and marginalized groups. While declines in mental health have occurred across the socioeconomic spectrum, the odds of poor/fair mental health were substantially higher among mothers who were single parents, younger, had less education, and those with multiracial, publicly insured, or uninsured children.

Research is urgently needed to identify the causes of lower and declining mental health among parents and to develop interventions that support prevention, diagnosis, and treatment of mental health conditions. Leading theories on the rise of mental health challenges in the US include limited access to mental health care, social isolation, rising substance use disorders, and broader stressors such as inflation, rising income inequality, racism, gun violence, and climate change.^5,30,31^ Among younger individuals, particularly members of Generation Z, it has further been posited that declining mental health may be related to recent shifts in childhood experiences (eg, exposure to social media during childhood and adolescence) and increased awareness and social acceptability of mental health concerns.^30,32,33^ Research elucidating the relative importance of different factors contributing to mental health declines among parents specifically could aid in evidence-based policy interventions to slow or reverse these declines.

Further, while concerns have been raised about rising levels of chronic conditions and obesity among reproductive-age and pregnant women,^34,35,36^ we found small declines in maternal physical health over the study period. However, disparities in self-reported physical health were notably larger than those for mental health, with lower physical health for racially minoritized groups, those with publicly insured children, less education, and single parents. Addressing the structural factors that drive inequities in physical diseases, including access to health care, exposures to social risks such as poverty, and the physical and built environment, should remain important targets for policy and public health intervention.^37^

Finally, we demonstrated that an existing national data resource, the NSCH, could serve as a monitoring tool for parental health. Adding additional variables, such as mental health diagnoses and/or symptoms through validated screening tools (eg, 4-item Patient Health Questionnaire), could further enhance the utility of these data for public health surveillance.

### Limitations

This study had limitations. First, health measures were limited to 2 single-item Likert response questions. While these simple self-reported health instruments are strong predictors of subsequent medical care and mortality, there is known reporting heterogeneity across sociodemographic groups, in particular by sex and age.^38^ We cannot separate true vs perceived health differences. Further, low self-reported health ratings do not translate into clinically meaningful diagnoses and may reflect more permanent/chronic aspects of health rather than acute conditions.^39^ Indeed, self-reported health measures tend to perform slightly worse than diagnostic measures at predicting short-term mortality but slightly better for long-term mortality.^39^ Despite these limitations, self-reported health does offer the advantage of not relying on health care use and thus is less affected by inequities in health care access across sociodemographic groups. Second, only the child’s, not the parent’s, race, ethnicity, and health insurance were measured in the NSCH; however, we included these child covariates as indicators of household socioeconomic status and exposure to structural racism and discrimination.^40^ Third, the sample size was small for some subgroups, for example, same-sex 2-parent households, limiting statistical power to examine trend differences and disparities relative to other groups.

## Conclusions

This cross-sectional analysis identified meaningful declines in self-reported maternal mental health from 2016 to 2023, while physical health declines were much smaller. As recently brought to attention by the 2024 Surgeon General’s advisory, addressing the rising population-level rates of poor maternal mental health both during and beyond the perinatal period should be a central focus of policy efforts to improve maternal and child health in the US.

## Notes

Supplement 1. eTable 1. Segmented regression analysis of self-reported maternal physical and mental health comparing levels and trends before (2016-2019) and during the COVID-19 pandemic (2020-2022) eFigure 1. Adjusted percent change in fair or poor self-reported maternal physical and mental health, 2016-2023 eTable 2. Trends in fair or poor self-reported maternal physical health by sample characteristics, 2016-2023 eTable 3. Trends in fair or poor maternal mental health by sample characteristics, 2016-2023 eTable 4. Sociodemographic characteristics associated with fair or poor self-reported maternal physical and mental health, 2016-2023 eFigure 2. Trends in self-reported paternal physical and mental health, 2016-2023 eTable 5. Annual trends and overall prevalence changes in self-reported paternal physical and mental health, 2016-2023 eTable 6. Segmented regression analysis of self-reported paternal physical and mental health comparing levels and trends before (2016-2019) and during the COVID-19 pandemic (2020-2022)

Supplement 2. Data Sharing Statement
