# Supplementary material for: Trends and Disparities in Maternal Self-Reported Mental and Physical Health
Source: JAMA Intern Med. 2025 May 27;185(7):857–65. doi: 10.1001/jamainternmed.2025.1260 (PMC12117492; doi:10.1001/jamainternmed.2025.1260)
Supplement: Supplement 1. — eTable 1. Segmented regression analysis of self-reported maternal physical and mental health comparing levels and trends before (2016-2019) and during the COVID-19 pandemic (2020-2022) eFigure 1. Adjusted percent change in fair or poor self-reported maternal physical and mental health, 2016-2023 eTable 2. Trends in fair or poor self-reported maternal physical health by sample characteristics, 2016-2023 eTable 3. Trends in fair or poor maternal mental health by sample characteristics, 2016-2023 eTable 4. Sociodemographic characteristics associated with fair or poor self-reported maternal physical and mental health, 2016-2023 eFigure 2. Trends in self-reported paternal physical and mental health, 2016-2023 eTable 5. Annual trends and overall prevalence changes in self-reported paternal physical and mental health, 2016-2023 eTable 6. Segmented regression analysis of self-reported paternal physical and mental health comparing levels and trends before (2016-2019) and during the COVID-19 pandemic (2020-2022) [file jamainternmed-e251260-s001.pdf]

Supplemental Online Content

Daw JR, MacCallum-Bridges CL, Admon LK. Trends and disparities in maternal self-reported mental and physical health. *JAMA Intern Med.* Published online May 27, 2025. doi:10.1001/jamainternmed.2025.1260

eTable 1. Segmented regression analysis of self-reported maternal physical and mental health comparing levels and trends before (2016-2019) and during the COVID-19 pandemic (2020-2022)

eFigure 1. Adjusted percent change in fair or poor self-reported maternal physical and mental health, 2016-2023

eTable 2. Trends in fair or poor self-reported maternal physical health by sample characteristics, 2016-2023

eTable 3. Trends in fair or poor maternal mental health by sample characteristics, 2016-2023

eTable 4. Sociodemographic characteristics associated with fair or poor self-reported maternal physical and mental health, 2016-2023

eFigure 2. Trends in self-reported paternal physical and mental health, 2016-2023

eTable 5. Annual trends and overall prevalence changes in self-reported paternal physical and mental health, 2016-2023

eTable 6. Segmented regression analysis of self-reported paternal physical and mental health comparing levels and trends before (2016-2019) and during the COVID-19 pandemic (2020-2022)

This supplemental material has been provided by the authors to give readers additional information about their work.

© 2025 Daw JR et al. *JAMA Internal Medicine*.

eTable 1 Segmented regression analysis of self-reported maternal physical and mental health comparing levels and trends before (2016-2019) and during the COVID-19 pandemic (2020-2022)

	Change in Prevalence Level Associated with the COVID-19 Pandemic		Change in Prevalence Trend Associated with the COVID-19 Pandemic	
	**Unadjusted**	**Adjusted[1]**	**Unadjusted**	**Adjusted[1]**
**Physical Health**				
Fair or poor	0.7 (-0.6, 2.0)	0.8 (-0.5, 2.1)	0.3 (-0.4, 0.9)	0.3 (-0.3, 0.9)
Good	-0.5 (-2.5, 1.6)	-0.3 (-2.4, 1.7)	-0.2 (-1.2, 0.8)	-0.2 (-1.2, 0.8)
Very good	0.4 (-1.8, 2.6)	0.3 (-1.8, 2.5)	0.6 (-0.5, 1.7)	0.6 (-0.5, 1.6)
Excellent	-0.6 (-2.6, 1.4)	-0.8 (-2.7, 1.2)	-0.7 (-1.6, 0.3)	-0.7 (-1.6, 0.2)
**Mental Health**				
Fair or poor	1.3 (0.2, 2.5)*	1.5 (0.3, 2.6)*	0.7 (0.1, 1.3)*	0.6 (0.0, 1.2)*
Good	0.6 (-1.3, 2.5)	0.8 (-1.0, 2.7)	0.1 (-0.8, 1.0)	0.1 (-0.8, 1.1)
Very good	-0.6 (-2.8, 1.5)	-0.7 (-2.8, 1.4)	-0.7 (-1.7, 0.4)	-0.7 (-1.7, 0.3)
Excellent	-1.3 (-3.4, 0.9)	-1.6 (-3.7, 0.5)	-0.1 (-1.2, 0.9)	-0.1 (-1.1, 0.9)

Notes: *Coefficient is statistically significant (i.e. different than zero), $p<0.05$. [1]Adjusted model includes child age, child race and ethnicity, child insurance status, maternal age, maternal education, maternal nativity, and family structure. Segmented regression analysis included a binary indicator for the COVID-19 pandemic years (2020-2022; representing the prevalence level change in the outcome associated with the pandemic), a linear yearly time trend (representing the pre-COVID annual trend in the outcome), and an interaction between the COVID-19 indicator and time trend (representing the change in the annual trend associated with the pandemic). The year 2023 was excluded from this analysis (i.e. not considered "during the COVID-19 pandemic") since the national public health emergency was declared over in 2023 and most restrictions had ended.

© 2025 Daw JR et al. *JAMA Internal Medicine*.

eFigure 1 Adjusted percent change in fair or poor self-reported maternal physical and mental health, 2016-2023

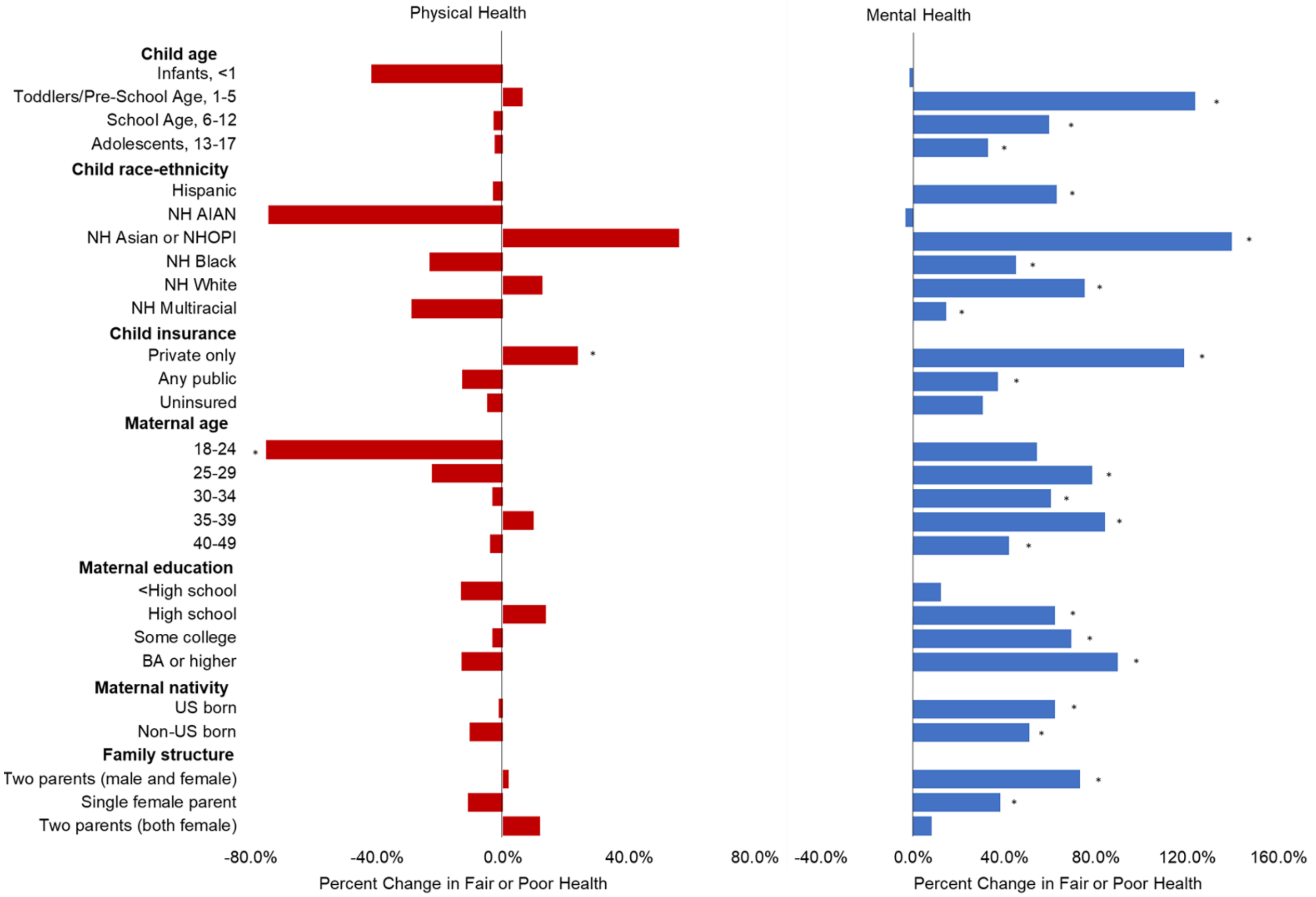

Notes: Percent change is calculated as the percentage point difference in the outcome from 2016-2023 divided by the baseline prevalence in 2016 multiplied by 100; *Percent change in maternal health from 2016 to 2023 is statistically significantly different from the null value (0) at the 0.05 significance level; NH = non-Hispanic, AIAN = American Indian or Alaska Native, NHOPI = Native Hawaiian or other Pacific Islander. Some college includes associate degree or vocational training. All estimates are presented in eTables 3-4.

© 2025 Daw JR et al. *JAMA Internal Medicine*.

eTable 2 Trends in fair or poor self-reported maternal physical health by sample characteristics, 2016-2023

	2016 Prevalence	2023 Prevalence	Prevalence Change, 2016-2023		Annual Trend, 2016-2023	
			Unadjusted	Adjusted[1]	Unadjusted	Adjusted[1]
**Child Age**						
Infants, <1	7.9 (4.3, 14.0)	2.9 (1.6, 5.2)	-4.9 (-10.0, 0.1)	-3.3 (-7.3, 0.8)	-0.4 (-0.9, 0.2)	-0.3 (-0.7, 0.2)
Toddlers/Pre-School Age, 1-5	5.0 (3.9, 6.3)	5.3 (4.4, 6.2)	0.3 (-1.2, 1.8)	0.3 (-1.2, 1.8)	0.2 (0.0, 0.4)	0.2 (0.0, 0.4)
School Age, 6-12	8.2 (7.0, 9.7)	7.5 (6.3, 8.9)	-0.7 (-2.6, 1.1)	-0.3 (-2.1, 1.6)	0.0 (-0.2, 0.2)	0.1 (-0.1, 0.3)
Adolescents, 13-17	10.2 (8.7, 12.0)	10.0 (8.7, 11.5)	-0.2 (-2.4, 1.9)	-0.3 (-2.4, 1.9)	0.0 (-0.2, 0.3)	0.0 (-0.2, 0.3)
**Child race and ethnicity**						
Hispanic	10.2 (8.2, 12.7)	9.7 (8.1, 11.7)	-0.5 (-3.4, 2.4)	-0.3 (-3.1, 2.5)	0.2 (-0.2, 0.5)	0.2 (-0.1, 0.5)
NH AIAN	12.0 (7.0, 19.7)	5.7 (2.7, 11.5)	-6.3 (-13.7, 1.1)	-8.9 (-18.0, 0.1)	-0.8 (-1.8, 0.3)	-0.9 (-2.0, 0.2)
NH Asian or NHOPI	4.2 (2.3, 7.5)	7.0 (4.9, 9.8)	2.8 (-0.7, 6.3)	2.3 (-1.4, 6.1)	0.4 (0.0, 0.8)*	0.4 (0.0, 0.8)
NH Black	13.9 (11.0, 17.4)	10.8 (8.6, 13.5)	-3.1 (-7.1, 0.9)	-3.3 (-7.3, 0.8)	-0.1 (-0.6, 0.3)	-0.1 (-0.5, 0.4)
NH White	5.2 (4.6, 5.8)	5.4 (4.8, 6.1)	0.2 (-0.7, 1.2)	0.6 (-0.2, 1.5)	0.1 (0.0, 0.2)	0.1 (0.0, 0.2)*
NH multiracial	9.8 (6.5, 14.5)	6.6 (5.3, 8.2)	-3.2 (-7.4, 1.0)	-2.8 (-6.9, 1.2)	-0.2 (-0.7, 0.2)	-0.3 (-0.7, 0.1)
**Child insurance status**						
Private	3.2 (2.7, 3.9)	3.8 (3.3, 4.3)	0.5 (-0.2, 1.3)	0.8 (0.0, 1.5)*	0.1 (0.0, 0.2)*	0.2 (0.1, 0.2)*
Any public	14.4 (12.6, 16.3)	13.0 (11.4, 14.8)	-1.3 (-3.8, 1.2)	-1.9 (-4.3, 0.6)	0.0 (-0.3, 0.3)	0.0 (-0.3, 0.3)
Uninsured	10.2 (7.0, 14.8)	10.1 (7.3, 14.0)	-0.1 (-5.2, 5.0)	-0.5 (-5.6, 4.6)	0.1 (-0.5, 0.7)	0.1 (-0.5, 0.6)
**Maternal age**						
18-24	10.3 (6.1, 16.8)	4.2 (2.1, 8.0)	-6.1 (-12, -0.2)*	-7.8 (-14.4, -1.1)*	-0.7 (-1.5, 0.0)	-0.9 (-1.8, -0.1)*
25-29	6.9 (5.0, 9.4)	5.3 (3.5, 8.1)	-1.5 (-4.6, 1.6)	-1.6 (-4.8, 1.7)	0.0 (-0.4, 0.3)	-0.1 (-0.4, 0.3)
30-34	7.1 (5.6, 9.1)	7.1 (5.7, 8.7)	-0.1 (-2.4, 2.2)	-0.2 (-2.5, 2.0)	0.1 (-0.2, 0.4)	0.1 (-0.2, 0.4)
35-39	7.3 (5.7, 9.2)	7.4 (6.0, 9.1)	0.2 (-2.2, 2.5)	0.7 (-1.5, 2.9)	0.2 (0.0, 0.5)*	0.3 (0.0, 0.5)*
≥40	8.5 (7.4, 9.9)	7.9 (7.0, 9.0)	-0.6 (-2.2, 1.0)	-0.4 (-1.9, 1.2)	0.0 (-0.2, 0.2)	0.0 (-0.1, 0.2)
**Maternal education**						
Less than high school	16.9 (13.3, 21.2)	15.8 (12.3, 20.0)	-1.1 (-6.7, 4.4)	-2.3 (-7.6, 3.1)	0.4 (-0.3, 1.0)	0.3 (-0.3, 1.0)
High school diploma	10.2 (8.5, 12.2)	11.9 (9.7, 14.6)	1.7 (-1.3, 4.8)	1.4 (-1.7, 4.4)	0.2 (-0.2, 0.5)	0.1 (-0.2, 0.4)
Some college	9.3 (7.8, 11.0)	9.5 (8.4, 10.8)	0.3 (-1.7, 2.2)	-0.3 (-2.2, 1.6)	0.1 (-0.1, 0.3)	0.0 (-0.2, 0.2)
BA or higher	3.2 (2.4, 4.1)	2.9 (2.5, 3.4)	-0.2 (-1.1, 0.7)	-0.4 (-1.3, 0.5)	0.1 (0.0, 0.2)	0.0 (0.0, 0.1)
**Maternal nativity**						
U.S. born	7.7 (6.9, 8.6)	7.3 (6.6, 8.1)	-0.4 (-1.6, 0.7)	-0.1 (-1.2, 1.0)	0.0 (-0.1, 0.1)	0.0 (-0.1, 0.2)
Non-U.S. born	8.3 (6.3, 10.8)	7.7 (6.2, 9.7)	-0.5 (-3.4, 2.3)	-0.9 (-3.7, 1.9)	0.3 (0.0, 0.6)	0.3 (-0.1, 0.6)
**Family structure**						
Two parents (different sex)	6.2 (5.3, 7.1)	5.9 (5.2, 6.7)	-0.3 (-1.4, 0.9)	0.1 (-1.0, 1.2)	0.1 (-0.1, 0.2)	0.1 (0.0, 0.2)
Single female parent	14.1 (12.1, 16.4)	12.5 (10.8, 14.5)	-1.6 (-4.4, 1.2)	-1.6 (-4.2, 1.1)	0.0 (-0.3, 0.3)	0.0 (-0.3, 0.3)
Two parents (same sex)	7.6 (3.3, 16.7)	8.5 (4.6, 15.2)	0.9 (-7.2, 9.0)	0.6 (-7.8, 9.0)	-0.4 (-1.5, 0.7)	-0.3 (-1.3, 0.8)

Notes: NH = non-Hispanic, AIAN = American Indian or Alaska Native, NHOPI = Native Hawaiian or other Pacific Islander. *Statistically significant, $p<0.05$.[1]Adjusted model includes child age, self-reported child race and ethnicity, child insurance status, maternal age, maternal education, maternal nativity, and family structure.

© 2025 Daw JR et al. *JAMA Internal Medicine*.

eTable 3 Trends in fair or poor maternal mental health by sample characteristics, 2016-2023

	2016 Prevalence	2023 Prevalence	Prevalence Change, 2016-2023		Annual Trend, 2016-2023	
			Unadjusted	Adjusted[1]	Unadjusted	Adjusted[1]
**Child Age**						
Infants, <1	8.0 (4.0, 15.5)	7.2 (4.8, 10.5)	-0.8 (-7.0, 5.3)	-0.1 (-5.4, 5.2)	0.0 (-0.7, 0.7)	0.1 (-0.6, 0.8)
Toddlers/Pre-School Age, 1-5	4.4 (3.7, 5.3)	9.4 (8.3, 10.7)	5.0 (3.5, 6.4)*	5.5 (4.0, 7.0)*	0.8 (0.6, 1.0)*	0.8 (0.7, 1.0)*
School Age, 6-12	5.6 (4.7, 6.7)	8.7 (7.5, 10.0)	3.1 (1.5, 4.7)*	3.3 (1.8, 4.9)*	0.6 (0.5, 0.8)*	0.7 (0.5, 0.9)*
Adolescents, 13-17	5.9 (4.9, 7.2)	7.6 (6.6, 8.8)	1.7 (0.1, 3.3)*	2.0 (0.4, 3.5)*	0.4 (0.2, 0.6)*	0.4 (0.2, 0.6)*
**Child race and ethnicity**						
Hispanic	5.1 (3.8, 7.0)	8.2 (6.8, 9.9)	3.1 (0.9, 5.3)*	3.2 (1.1, 5.3)*	0.6 (0.3, 0.8)*	0.6 (0.3, 0.8)*
NH AIAN	6.1 (2.9, 12.3)	4.5 (1.6, 11.8)	-1.6 (-7.9, 4.6)	-0.2 (-7.3, 6.9)	0.0 (-0.8, 0.8)	0.2 (-0.7, 1.2)
NH Asian or NHOPI	2.6 (1.0, 6.4)	6.5 (3.9, 10.5)	4.0 (0.0, 7.9)	3.6 (-0.3, 7.4)	0.5 (0.1, 1.0)*	0.5 (0.1, 0.9)*
NH Black	8.7 (6.5, 11.6)	12.0 (9.8, 14.7)	3.3 (-0.2, 6.8)	3.9 (0.4, 7.5)*	0.6 (0.2, 1.1)*	0.7 (0.3, 1.1)*
NH White	4.8 (4.2, 5.4)	7.8 (7.1, 8.6)	3.0 (2.1, 4.0)*	3.6 (2.6, 4.5)*	0.6 (0.5, 0.7)*	0.7 (0.5, 0.8)*
NH multiracial	9.1 (5.8, 14.0)	10.1 (7.8, 13)	1.0 (-3.8, 5.8)	1.3 (-3.3, 5.9)	0.5 (0.0, 1.0)*	0.5 (0.0, 1.0)*
**Child insurance status**						
Private	2.9 (2.4, 3.5)	6.0 (5.4, 6.7)	3.1 (2.3, 4.0)*	3.4 (2.6, 4.3)*	0.5 (0.4, 0.6)*	0.5 (0.4, 0.6)*
Any public	9.3 (7.9, 10.8)	12.7 (11.2, 14.3)	3.4 (1.3, 5.5)*	3.4 (1.3, 5.5)*	0.8 (0.6, 1.0)*	0.8 (0.6, 1.0)*
Uninsured	6.6 (4.6, 9.5)	8.6 (6.4, 11.4)	2.0 (-1.5, 5.4)	2.0 (-1.4, 5.4)	0.4 (-0.1, 0.9)	0.4 (-0.1, 0.9)
**Maternal age**						
18-24	8.5 (5.6, 12.9)	13.6 (8.8, 20.5)	5.1 (-1.7, 11.9)	4.6 (-2.4, 11.7)	0.9 (0.1, 1.7)*	0.8 (-0.1, 1.6)
25-29	6.5 (4.9, 8.5)	10.7 (8.5, 13.5)	4.3 (1.2, 7.3)*	5.1 (2.0, 8.2)*	1.0 (0.6, 1.4)*	1.1 (0.7, 1.5)*
30-34	6.4 (5.0, 8.3)	10.8 (9.3, 12.4)	4.3 (2.1, 6.5)*	3.9 (1.7, 6.1)*	0.8 (0.5, 1.1)*	0.8 (0.5, 1.1)*
35-39	5.4 (4.2, 6.9)	9.6 (8.0, 11.4)	4.2 (2.0, 6.4)*	4.5 (2.4, 6.7)*	0.7 (0.5, 1.0)*	0.8 (0.5, 1.0)*
≥40	4.7 (3.9, 5.7)	6.6 (5.8, 7.5)	1.9 (0.7, 3.2)*	2.0 (0.8, 3.2)*	0.4 (0.2, 0.5)*	0.4 (0.2, 0.5)*
**Maternal education**						
Less than high school	8.9 (6.6, 11.9)	10.2 (7.5, 13.6)	1.3 (-2.7, 5.3)	1.1 (-2.9, 5.1)	0.4 (-0.1, 0.9)	0.4 (-0.1, 0.9)
High school diploma	7.2 (5.6, 9.2)	11.8 (9.8, 14.3)	4.6 (1.7, 7.5)*	4.5 (1.7, 7.3)*	0.9 (0.6, 1.3)*	0.9 (0.6, 1.2)*
Some college	6.8 (5.6, 8.2)	11.3 (10.0, 12.7)	4.6 (2.7, 6.4)*	4.7 (2.8, 6.5)*	0.8 (0.6, 1.0)*	0.8 (0.5, 1.0)*
BA or higher	2.9 (2.4, 3.6)	5.6 (4.9, 6.3)	2.6 (1.7, 3.6)*	2.6 (1.7, 3.6)*	0.5 (0.4, 0.6)*	0.5 (0.4, 0.6)*
**Maternal nativity**						
U.S. born	6.2 (5.5, 6.9)	9.6 (8.8, 10.4)	3.4 (2.3, 4.5)*	3.8 (2.8, 4.9)*	0.6 (0.5, 0.8)*	0.7 (0.6, 0.8)*
Non-U.S. born	3.1 (1.9, 4.8)	4.8 (3.8, 6.0)	1.7 (-0.1, 3.5)	1.6 (-0.2, 3.3)	0.4 (0.1, 0.6)*	0.3 (0.1, 0.6)*
**Family structure**						
Two parents (different sex)	4.5 (3.8, 5.2)	7.3 (6.6, 8.1)	2.8 (1.9, 3.8)*	3.3 (2.3, 4.2)*	0.6 (0.4, 0.7)*	0.6 (0.5, 0.7)*
Single female parent	9.5 (7.8, 11.4)	12.6 (10.9, 14.5)	3.1 (0.6, 5.6)*	3.6 (1.2, 6.0)*	0.6 (0.3, 1.0)*	0.7 (0.4, 1.0)*
Two parents (same sex)	5.0 (1.7, 13.6)	5.8 (3.2, 10.3)	0.8 (-5.4, 7.0)	0.4 (-6.0, 6.8)	0.1 (-0.8, 1.0)	0.0 (-0.9, 0.9)

Notes: NH = non-Hispanic, AIAN = American Indian or Alaska Native, NHOPI = Native Hawaiian or other Pacific Islander. *Statistically significant, $p<0.05$.[1]Adjusted model includes child age, self-reported child race and ethnicity, child insurance status, maternal age, maternal education, maternal nativity, and family structure.

© 2025 Daw JR et al. *JAMA Internal Medicine*.

eTable 4 Sociodemographic characteristics associated with fair or poor self-reported maternal physical and mental health, 2016-2023

	**Pooled Prevalence 2016-2023**	**Unadjusted Odds Ratio**	**Adjusted[1] Odds Ratio**
**Physical Health**	**% (95% CI)**	**OR (95% CI)**	**aOR (95% CI)**
**Child Age**			
<1	4.2 (3.1, 5.5)	0.54 (0.40, 0.78)*	0.68 (0.50, 0.92)*
1-5	5.2 (4.7, 5.8)	0.69 (0.61, 0.78)*	0.81 (0.71, 0.92)*
6-12	7.4 (7.0, 7.9)	Reference	Reference
13-17	9.6 (9.0, 10.2)	1.32 (1.20, 1.46)*	1.18 (1.06, 1.31)*
**Child race and ethnicity**			
Hispanic	9.6 (8.8, 10.4)	2.04 (1.84, 2.27)*	1.36 (1.21, 1.54)*
NH AIAN	10.7 (8.1, 14.0)	2.30 (1.68, 3.14)*	1.45 (1.03, 2.02)*
NH Asian or NHOPI	5.3 (4.3, 6.4)	1.07 (0.87, 1.33)	1.33 (1.05, 1.69)*
NH Black	11.8 (10.8, 12.9)	2.58 (2.30, 2.88)*	1.42 (1.25, 1.62)*
NH White	4.9 (4.7, 5.2)	Reference	Reference
NH multiracial	8.2 (7.3, 9.2)	1.72 (1.49, 1.98)*	1.54 (1.33, 1.78)*
**Child insurance status**			
Private	3.4 (3.2, 3.6)	Reference	Reference
Any public	13.2 (12.5, 13.9)	4.28 (3.93, 4.66)*	2.62 (2.35, 2.91)*
Uninsured	8.9 (7.6, 10.5)	2.76 (2.28, 3.34)*	1.67 (1.37, 2.03)*
**Maternal age**			
18-24	7.4 (5.5, 10.1)	0.96 (0.69, 1.35)	0.56 (0.39, 0.79)*
25-29	6.7 (5.8, 7.8)	0.86 (0.72, 1.03)	0.59 (0.49, 0.73)*
30-34	6.9 (6.3, 7.7)	0.89 (0.79, 1.01)	0.76 (0.66, 0.88)*
35-39	6.9 (6.3, 7.4)	0.88 (0.79, 0.98)*	0.85 (0.76, 0.95)*
≥40	7.7 (7.3, 8.2)	Reference	Reference
**Maternal education**			
Less than high school	15.4 (13.8, 17.0)	6.33 (5.49, 7.30)*	3.66 (3.11, 4.30)*
High school diploma	10.5 (9.8, 11.4)	4.11 (3.68, 4.60)*	2.47 (2.18, 2.81)*
Some college	9.2 (8.6, 9.7)	3.52 (3.19, 3.89)*	2.35 (2.11, 2.62)*
Bachelor's degree or more	2.8 (2.6, 3.0)	Reference	Reference
**Maternal nativity**			
U.S. born	7.1 (6.9, 7.4)	Reference	Reference
Non-U.S. born	7.7 (6.9, 8.6)	1.09 (0.96, 1.23)	0.58 (0.50, 0.67)*
**Family structure**			
Two parents (different sex)	5.5 (5.3, 5.9)	Reference	Reference
Single female parent	12.8 (12.1, 13.6)	2.51 (2.29, 2.74)*	1.45 (1.31, 1.61)*
Two parents (same sex)	9.9 (7.4, 13.2)	1.87 (1.35, 2.60)*	1.57 (1.12, 2.21)*
**Mental Health**	**% (95% CI)**	**OR (95% CI)**	**aOR (95% CI)**
**Child Age**			
<1	6.2 (4.9, 8)	0.91 (0.69, 1.20)	0.87 (0.65, 1.16)
1-5	7.0 (6.6, 7.5)	1.04 (0.94, 1.14)	1.00 (0.90, 1.12)
6-12	6.8 (6.4, 7.2)	Reference	Reference
13-17	7.1 (6.7, 7.6)	1.05 (0.96, 1.16)	1.09 (0.98, 1.21)
**Child race and ethnicity**			
Hispanic	7.0 (6.4, 7.7)	1.18 (1.05, 1.31)*	1.01 (0.89, 1.15)
NH AIAN	7.0 (5.0, 9.7)	1.17 (0.82, 1.67)	0.80 (0.56, 1.14)
NH Asian or NHOPI	3.7 (2.9, 4.6)	0.60 (0.47, 0.76)*	0.94 (0.72, 1.22)
NH Black	10.0 (9.0, 11.0)	1.72 (1.53, 1.94)*	1.10 (0.96, 1.25)
NH White	6.0 (5.8, 6.3)	Reference	Reference
NH multiracial	9.9 (8.9, 11.1)	1.71 (1.51, 1.95)*	1.55 (1.35, 1.77)*
**Child insurance status**			
Private	4.4 (4.2, 4.7)	Reference	Reference

© 2025 Daw JR et al. *JAMA Internal Medicine*.

Any public	10.8 (10.3, 11.4)	2.62 (2.41, 2.84)*	1.92 (1.73, 2.12)*
Uninsured	7.7 (6.5, 9.1)	1.80 (1.48, 2.18)*	1.44 (1.18, 1.75)*
**Maternal age**			
18-24	12.5 (10.0, 15.4)	2.31 (1.79, 2.96)*	1.37 (1.04, 1.80)*
25-29	9.1 (8.2, 10.1)	1.63 (1.43, 1.86)*	1.15 (0.99, 1.35)
30-34	8.2 (7.5, 8.9)	1.44 (1.29, 1.61)*	1.24 (1.10, 1.41)*
35-39	6.9 (6.4, 7.4)	1.20 (1.08, 1.33)*	1.15 (1.04, 1.28)*
≥40	5.8 (5.5, 6.2)	Reference	Reference
**Maternal education**			
Less than high school	10.0 (8.8, 11.3)	2.53 (2.18, 2.95)*	1.91 (1.61, 2.27)*
High school diploma	9.4 (8.7, 10.2)	2.37 (2.12, 2.65)*	1.55 (1.37, 1.76)*
Some college	8.6 (8.1, 9.1)	2.15 (1.96, 2.36)*	1.52 (1.37, 1.68)*
Bachelor's degree or more	4.2 (3.9, 4.5)	Reference	Reference
**Maternal nativity**			
U.S. born	7.5 (7.3, 7.8)	Reference	Reference
Non-U.S. born	4.7 (4.1, 5.3)	0.60 (0.52, 0.69)*	0.47 (0.40, 0.56)*
**Family structure**			
Two parents (different sex)	5.6 (5.3, 5.8)	Reference	Reference
Single female parent	11.4 (10.7, 12.2)	2.19 (2.01, 2.39)*	1.58 (1.44, 1.74)*
Two parents (same sex)	7.9 (5.7, 10.9)	1.46 (1.03, 2.09)*	1.38 (0.96, 1.98)

Notes: NH = non-Hispanic, AIAN = American Indian or Alaska Native, NHOPI = Native Hawaiian or other Pacific Islander. Some college includes associate degree or vocational training.*p<0.05 relative to reference group; [1]Adjusted models include year fixed effects and all other listed characteristics (i.e., child age, self-reported child race and ethnicity, child insurance status, maternal age, maternal education, maternal nativity, and family structure)

© 2025 Daw JR et al. *JAMA Internal Medicine*.

eFigure 2 Trends in categorical Likert responses for self-reported paternal physical and mental health , 2016-2023

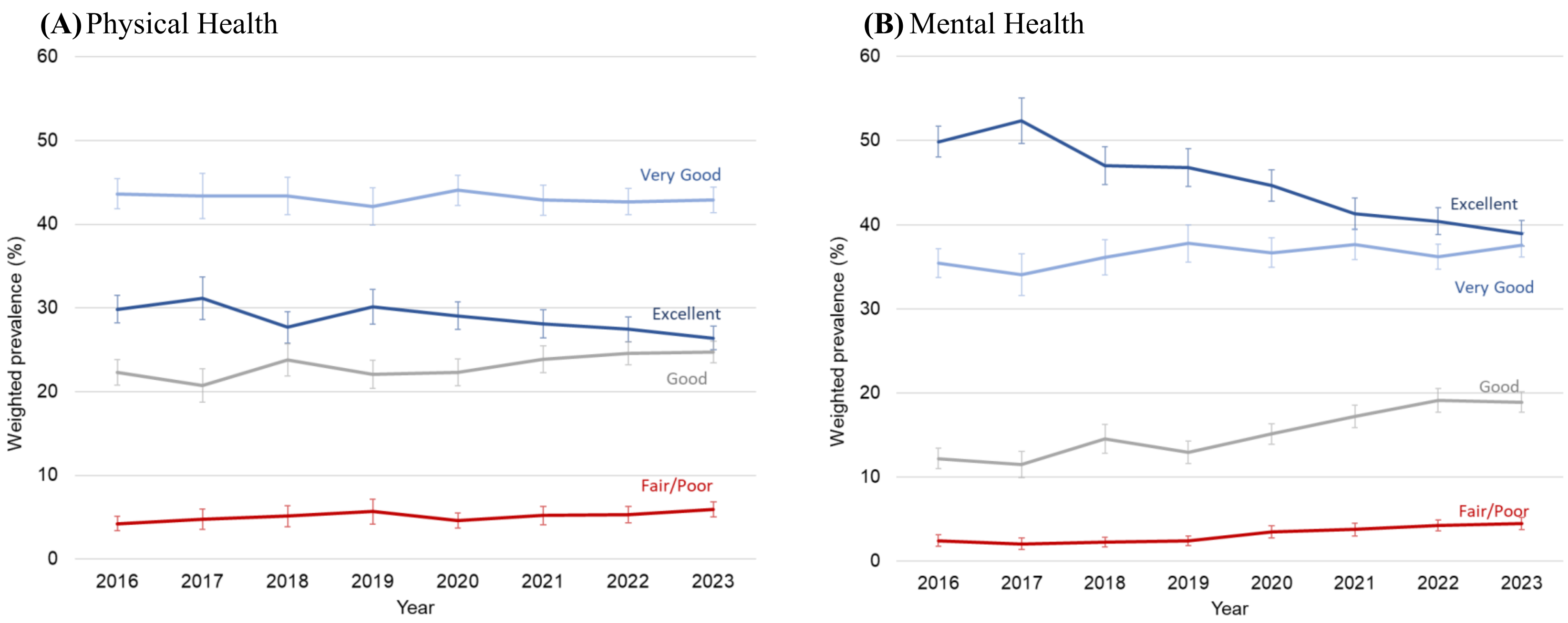

© 2025 Daw JR et al. *JAMA Internal Medicine*.

eTable 5 Trends in self-reported paternal physical and mental health, 2016-2023

	2016 Prevalence, % (95% CI)	2023 Prevalence, % (95% CI)	Annual Trend, 2016-2023		Prevalence Change, 2016-2023		
			Unadjusted, PP (95% CI)	Adjusted[1], PP (95% CI)	Unadjusted, PP (95% CI)	Adjusted[1], PP (95% CI)	Adjusted Percent Change from 2016, %
**Physical Health**							
Fair or poor	4.2 (3.6, 5.0)	5.9 (5.2, 6.8)	0.2 (0.0, 0.3)*	0.2 (0.0, 0.3)*	1.7 (0.6, 2.8)*	1.8 (0.7, 2.9)*	42.8
Good	22.3 (20.7, 23.9)	24.8 (23.5, 26.1)	0.4 (0.2, 0.7)*	0.4 (0.2, 0.7)*	2.5 (0.4, 4.5)*	3.0 (0.9, 5.1)*	13.4
Very Good	43.6 (41.8, 45.4)	42.9 (41.4, 44.4)	-0.1 (-0.4, 0.2)	-0.1 (-0.4, 0.2)	-0.7 (-3.1, 1.6)	-0.9 (-3.3, 1.5)	-2.1
Excellent	29.9 (28.2, 31.6)	26.4 (25.0, 27.9)	-0.5 (-0.8, -0.2)*	-0.5 (-0.8, -0.2)*	-3.4 (-5.7, -1.2)*	-3.9 (-6.1, -1.7)*	-13.0
**Mental Health**							
Fair or poor	2.4 (1.9, 3.1)	4.5 (3.9, 5.1)	0.4 (0.3, 0.5)*	0.4 (0.3, 0.5)*	2.1 (1.2, 2.9)*	2.0 (1.1, 2.8)*	83.3
Good	12.2 (11.0, 13.5)	18.9 (17.7, 20.2)	1.1 (0.9, 1.4)*	1.1 (0.9, 1.4)*	6.7 (4.9, 8.5)*	6.7 (5.0, 8.5)*	54.9
Very Good	35.4 (33.7, 37.2)	37.6 (36.1, 39.1)	0.3 (0.1, 0.6)*	0.3 (0.1, 0.6)*	2.1 (-0.1, 4.4)	1.8 (-0.5, 4.1)	5.1
Excellent	49.9 (48.0, 51.7)	39.0 (37.4, 40.5)	-1.9 (-2.2, -1.6)*	-1.9 (-2.2, -1.6)*	-10.9 (-13.3, -8.5)*	-10.5 (-12.8, -8.1)*	-21.0

Notes: *Change or annual trend is statistically different than zero, $p<0.05$.[1]Adjusted model includes child age, self-reported child race and ethnicity, child insurance status, paternal age, paternal education, paternal nativity, and family structure. Total sample size for male parents is N=91,435.

© 2025 Daw JR et al. *JAMA Internal Medicine*.

eTable 6 Segmented regression analysis of self-reported paternal physical and mental health comparing levels and trends before (2016-2019) and during the COVID-19 pandemic (2020-2022)

	**Change in Prevalence Level Associated with the COVID-19 Pandemic**		**Change in Prevalence Trend Associated with the COVID-19 Pandemic**	
	**Unadjusted**	**Adjusted[1]**	**Unadjusted**	**Adjusted[1]**
**Physical Health**				
Fair or poor	-1.4 (-3.1, 0.2)	-1.5 (-3.0, 0.1)	-0.1 (-0.9, 0.6)	-0.2 (-0.9, 0.6)
Good	-0.4 (-3.0, 2.3)	-0.2 (-2.7, 2.4)	0.9 (-0.4, 2.2)	1.0 (-0.3, 2.3)
Very good	1.9 (-1.2, 5.0)	1.8 (-1.3, 4.9)	-0.2 (-1.7, 1.3)	-0.3 (-1.8, 1.2)
Excellent	-0.1 (-3.0, 2.8)	-0.2 (-3.1, 2.7)	-0.6 (-2.0, 0.9)	-0.5 (-1.9, 0.8)
**Mental Health**				
Fair or poor	1.1 (0.2, 2.0)*	1.1 (0.2, 2.0)*	0.3 (-0.2, 0.9)	0.4 (-0.1, 0.9)
Good	1.1 (-1.1, 3.2)	1.0 (-1.0, 3.1)	1.5 (0.3, 2.6)*	1.6 (0.5, 2.7)*
Very good	-1.0 (-4.1, 2.1)	-1.1 (-4.2, 1.9)	-1.2 (-2.6, 0.3)	-1.1 (-2.6, 0.3)
Excellent	-1.1 (-4.3, 2.0)	-1.0 (-4.2, 2.1)	-0.7 (-2.2, 0.9)	-0.9 (-2.4, 0.6)

Notes: *Coefficient is statistically significant (i.e. different than zero), $p<0.05$. [1]Adjusted model includes child age, child race and ethnicity, child insurance status, paternal age, paternal education, paternal nativity, and family structure. Segmented regression analysis included a binary indicator for the COVID-19 pandemic years (2020-2022; representing the prevalence level change in the outcome associated with the pandemic), a linear yearly time trend relative to the start of the COVID-19 pandemic (representing the pre-COVID annual trend in the outcome), and an interaction between the COVID-19 indicator and time trend (representing the change in the annual trend associated with the pandemic). The year 2023 was excluded from this analysis (i.e. not considered “during the COVID-19 pandemic”) since the national public health emergency was declared over in 2023 and most restrictions had ended.

© 2025 Daw JR et al. *JAMA Internal Medicine*.
