# Supplementary material for: Trends and Disparities in Maternal Self-Reported Mental and Physical Health
Source: JAMA Intern Med. 2025 May 27;185(7):857–65. doi: 10.1001/jamainternmed.2025.1260 (PMC12117492; doi:10.1001/jamainternmed.2025.1260)
Supplement: Supplement 2. — Data Sharing Statement [file jamainternmed-e251260-s002.pdf]

Data Sharing Statement

Daw. Trends and Disparities in Maternal Self-Reported Mental and Physical Health. *JAMA Intern Med*. Published May 27, 2025. doi:10.1001/jamainternmed.2025.1260

Data
Data available: Yes
Data types: Data (not involving human participants), Data dictionary
How to access data: The data used in this study is publicly available for download from the U.S. Census Bureau: https://www.census.gov/programs-surveys/nsch.html.
When available: With publication

Supporting Documents
Document types: Statistical/analytic code
How to access documents: Requests for statistical code can be sent to Colleen MacCallum-Bridges, maccallc@med.umich.edu.
When available: With publication

Additional Information
Who can access the data: Anyone requesting the data or statistical code.
Types of analyses: For any purpose.
Mechanisms of data availability: The data is publicly available and provided by the U.S. Census Bureau. The supporting statistical code will be sent without an agreement upon individual request.
